# Hospital Formula Supplementation Postbreastfeeding Initiation, Neighborhood Economy, and Race

**DOI:** 10.1001/jamapediatrics.2025.5379

**Published:** 2025-12-29

**Authors:** Alison Mildon, Gillian D. Alton, Jo-Anna B. Baxter, Bronwyn Underhill, Daniel W. Sellen, Deborah L. O’Connor

**Affiliations:** 1 Nutritional Sciences, Temerty Faculty of Medicine, University of Toronto, Toronto, Ontario, Canada; 2 BORN Ontario, Children’s Hospital of Eastern Ontario, Ottawa, Ontario, Canada; 3 Parkdale Queen West Community Health Centre, Toronto, Ontario, Canada; 4 Joannah and Brian Lawson Centre for Child Nutrition, University of Toronto, Toronto, Ontario, Canada; 5 Translational Medicine Program, The Hospital for Sick Children, Toronto, Ontario, Canada

## Abstract

**Importance:**

Breastfeeding supports lifelong health, but socioeconomic and racial disparities persist. Biases in hospital formula supplementation practices may be an underlying contributor.

**Objective:**

To examine whether nonmedically indicated hospital formula supplementation of term-born breastfed newborns is associated with neighborhood socioeconomic status and/or maternal race.

**Design, Setting, and Participants:**

Provincial registry data were used to build a cohort of all live births of term-born singleton infants who initiated breastfeeding in Ontario, Canada, hospitals from April 1, 2015 through March 31, 2021, and for whom prenatal screening data were available. Of 570 936 eligible births, 148 888 were excluded, primarily due to missing outcome data, preterm birth, or not initiating breastfeeding. These data were analyzed from December 2023 through October 2025.

**Exposures:**

The 2 exposures were socioeconomic status, derived by linking maternal postal codes with 2021 Ontario Marginalization Index neighborhood-level quintiles for material resources, and maternal race (Asian, Black, White, or other [Indigenous, multiracial, or unknown race]), determined from prenatal screening data.

**Main Outcome and Measure:**

The primary outcome was nonmedically indicated formula supplementation, determined from hospital feeding records.

**Results:**

This cohort included 422 048 maternal-infant dyads, 28% of whom were in the Asian racial group, 7% in the Black racial group, 59% in the White racial group, and 5% in the other racial group. Overall, 27% of infants received nonmedically indicated formula supplementation, with an increase from 23% to 32% over the study period. Participants in the Asian, Black, and other racial groups were more likely than those in the White group to be in the most marginalized socioeconomic quintile (20%, 43%, and 23% vs 16%). Risk of nonmedically indicated formula supplementation increased in a gradient across quintiles of increasing socioeconomic marginalization (quintile 5 vs quintile 1: adjusted relative risk [aRR], 1.68; 95% CI, 1.64-1.72) and was significantly elevated for the Asian (aRR, 2.69; 95% CI, 2.64-2.74), Black (aRR, 2.07; 95% CI, 2.01-2.13), and other (aRR, 1.43; 95% CI, 1.39-1.48) racial groups compared with the White group.

**Conclusions and Relevance:**

In this population-level analysis, nonmedically indicated formula supplementation prevalence was high and increased over time, with elevated risk associated with socioeconomic marginalization and maternal racialization. Increased hospital adherence to breastfeeding support guidelines is needed to improve health equity.

## Key Points

**Question:**

Is neighborhood-level socioeconomic status and/or maternal race associated with increased risk of nonmedically indicated supplementation of breastfed newborns during the postpartum hospital stay?

**Findings:**

Using population-based registry data (2015-2021) for 422 048 term-born breastfed infants, 27% received nonmedically indicated formula in hospital, with a gradient of increasing risk across quintiles of increasing socioeconomic marginalization, and significantly increased risk for the Asian, Black, and other (Indigenous, multiracial, or unknown race) racial groups compared with the White group.

**Meaning:**

Improved adherence to established global guidelines supporting exclusive breastfeeding in birth hospitals holds potential to reduce breastfeeding disparities and improve health equity.

## Teaser

This study examines whether nonmedically indicated hospital formula supplementation of term-born breastfed newborns is associated with neighborhood socioeconomic status and/or maternal race.

## Introduction

Given associations between breast milk and optimal infant maturation and lifelong health, breastfeeding is recommended exclusively for the first 6 months then continued alongside complementary feeding to age 2 years and beyond.^1,2,3^ Canada has a publicly funded health care system and high breastfeeding initiation rates (91%), but over 40% of mothers discontinue in the early postpartum months and only 35% exclusively breastfeed for 6 months.^4^ There are also persistent sociodemographic disparities, including lower breastfeeding rates among mothers with lower incomes.^4,5^ Racial disparities in breastfeeding practices are not well studied in Canada, but are reported in the US.^6^

Determinants of breastfeeding practices are complex and operate at multiple levels.^7^ The key driver at the birth hospitalization level is the degree of implementation of the Ten Steps to Successful Breastfeeding defined by the global Baby-Friendly Initiative (BFI).^8^ This article focuses on supplementation practices (step 6). Providing breast milk substitutes (ie, infant formula, most of which is nonmedically indicated) during the postpartum hospital stay is an established risk factor for early breastfeeding cessation.^9,10,11^ Unnecessary supplementation interferes with the establishment of lactation by reducing the frequency of feeding at the breast, and consequently, milk production, and undermines mothers’ confidence in their breast milk supply, often by misinterpreting normal newborn behaviors as signs of hunger.^12,13,14,15^ Canadian neonatal care guidelines align with the Ten Steps,^3^ yet the most recent BFI scorecard (2019) for the province of Ontario reported that only 8 of 91 hospitals were BFI certified and 28% of newborns received in-hospital formula supplementation without medical reason.^16^

There is also evidence of supplementation disparities in Canada, including higher rates among mothers with lower socioeconomic status.^17,18^ This warrants examination on a population level with a specific focus on nonmedically indicated supplementation. Data from the US show racial disparities in hospital formula supplementation,^19^ but these are reduced by strengthening adherence to BFI principles.^20^ Given differing demographic and health system contexts, there is a need to examine potential racial disparities in supplementation practices in Canada. Therefore, we conducted a population-based study to examine associations between maternal socioeconomic status and race and nonmedically indicated supplementation of term-born infants who initiated breastfeeding in Ontario, Canada.

## Methods

### Study Design and Population

We used data from the Better Outcomes Registry & Network (BORN), a prescribed registry authorized to manage routine collection of antenatal, delivery, and newborn data on all births (140 000 per year) across the province of Ontario.^21^ The study population was extracted from the BORN data for all singleton live births in Ontario hospitals between April 1, 2015, and March 31, 2021, with prenatal screening data available. We included maternal-infant records for term births (gestational age, ≥37 weeks) above the low birth weight cutoff (≥2500 g) who initiated breastfeeding. Exclusion criteria were birth outside of a hospital, missing hospital feeding data, infant discharge to social services, infant adoption, surrogate birth, and/or postpartum transfer to another hospital. We followed Strengthening the Reporting of Observational Studies in Epidemiology (STROBE) reporting guideline using Observational Routinely-Collected Health Data (RECORD).^22^

### Ethics Approval

This study used routinely collected data by province-wide registries; no additional data were collected from patients. The study protocol was approved by the Office of Research Ethics at the University of Toronto (protocol 44917) and the Children’s Hospital of Eastern Ontario (protocol 23/03PE) and waived informed consent.

### Study Variables

The primary outcome was nonmedically indicated supplementation during the postpartum hospital stay. BORN feeding data are extracted from medical records by trained personnel and recorded as supplement use (yes/no) and the reason. According to BORN, infant medical reasons for supplementation are defined as formula ordered by a physician or nurse practitioner to address hypoglycemia, inadequate weight gain, inborn errors of metabolism, significant weight loss, or other clinical indicators (specification not required). Maternal medical reasons include active herpes on breast, contraindicated medications, HIV, severe maternal illness preventing breast milk feeding, and other health concerns resulting in a physician or nurse practitioner ordering formula.

The 2 exposure variables were maternal socioeconomic status and race, which we considered proxies for the social processes of socioeconomic marginalization and racialization. Socioeconomic status was determined at the neighborhood level by linking maternal postal codes with the 2021 Ontario Marginalization Index (ON-Marg) quintiles for material resources. ON-Marg integrates 6 income-related and asset-related indicators, such as unemployment and education levels, from 2021 national census data into an area-level measure of socioeconomic position.^23^

Maternal race data were extracted from the provincial prenatal screening form submitted by primary antenatal health care professionals, with 4 groupings: Asian, Black, White, and other. BORN analyses group Indigenous identity in the other group as the principles of Indigenous data sovereignty cannot be fulfilled within a provincial-level registry. The other group also includes multiracial individuals or unknown race.

Covariates for analyses of associations between our exposure variables and primary outcome were selected based on the literature and availability in the BORN dataset.^7^ Sociodemographic characteristics included maternal age at time of birth in years (categorized as 15-19, 20-24, 25-29, 30-34, 35-39, and ≥40); birth hospital region, classified by the first letter of the postal code (K: Eastern Ontario, L: Central Ontario, M: Metropolitan Toronto, N: Southwestern Ontario, P: Northern Ontario); rural residence, derived from maternal postal code; and multiparity. Maternal health characteristics included prepregnancy body mass index ([BMI] calculated as weight in kilograms divided by height in meters squared) (categorized as <18.5, 18.5-24.9, 25.0-29.9, 30.0-34.9, and >35); preexisting health conditions; mental health concerns; and smoking, substance use, and cannabis use during pregnancy.

The following perinatal characteristics were also collected for exploratory analysis: primary antenatal health care professional (physician, midwife, other, and shared care), prenatal intention to breastfeed (no, yes, unsure), study year based on infant date of birth, mode of delivery (vaginal, cesarean birth), gestational age at birth based on completed weeks of gestation (categorized as 37-38, 39-40, 41, and ≥42), birth weight in grams (categorized as 2500-3999, 4000-4500, and >4500), sex assigned at birth (female, male), neonatal intensive care admission, skin-to-skin contact within 2 hours of birth, infant positioned to breastfeed within 2 hours of birth, and provision of breastfeeding education and support within 6 hours of birth.

### Statistical Analyses

Descriptive statistics were performed for all variables and reported as counts and frequencies. Standardized differences were used to compare characteristics between socioeconomic quintiles 2 through 5 and quintile 1, and between the Asian, Black, and other racial groups and the White group; differences more than 0.10 indicate meaningful disparities.^24^

Multivariable logistic regression analyses were conducted to examine the association between the exposure variables (maternal socioeconomic status, maternal race) and the outcome of nonmedically indicated supplementation (yes/no), controlling for covariates. Variables were added in a step-wise fashion. Model 1 adjusted for maternal sociodemographics (age, ON-Marg quintile or race, rural residence, birth hospital region, parity). Model 2 adjusted for model 1 covariates and maternal health characteristics (prepregnancy BMI, preexisting health conditions, mental health concerns, prenatal smoking, prenatal substance use, prenatal cannabis use). Imputation was not used; missing data were excluded from analysis. Adjusted relative risks (aRR) with 95% CIs were calculated to assess differences in nonmedically indicated hospital formula supplementation between socioeconomic quintiles 2 through 5 compared with quintile 1 (least marginalized; reference group), and among Asian, Black, and other racial groups compared with the White group (reference group). Models were compared using the Akaike information criterion (AIC) value; the lowest AIC value was deemed to fit best.^25^

We also conducted subgroup analyses to investigate differences in associations between maternal socioeconomic status or race and nonmedically indicated supplementation before and during the COVID-19 pandemic by stratifying the regression models to compare study years 1 through 5 (April 1, 2015-March 31, 2020) with year 6 (April 1, 2020-March 31, 2021). Lastly, we conducted preplanned exploratory analyses examining the association between our exposure variables and nonmedically indicated formula supplementation, adjusted for the model 2 covariates and perinatal characteristics listed above. Analyses were conducted using SAS version 9.4 (SAS Institute).

## Results

The study cohort included 422 048 mother-infant dyads, 74% of the total singleton live births with prenatal screening data (eFigure in Supplement 1). Those ineligible due to missing prenatal screening data were more likely to live in rural areas, to be multiparous, and to smoke during pregnancy, and were less likely to intend to breastfeed (eTable 1 in Supplement 1). Characteristics of the cohort are presented by maternal socioeconomic status (Table 1) and race (Table 2). Compared with socioeconomic quintile 1 (least marginalized), participants living in more marginalized neighborhoods were more likely to be younger, to be in the Asian or Black racial groups, to give birth at hospitals in metropolitan Toronto, to smoke during pregnancy, to have a physician as primary antenatal health care professional, and to receive postpartum breastfeeding support in hospital. They were less likely to have a prepregnancy BMI in the recommended range (18.5-24.9) or to have a midwife as a primary health care professional. Those in quintile 5 were more likely to be multiparous and to use cannabis during pregnancy. Compared with the White racial group, participants in the Asian, Black, and other groups were more likely to live in socioeconomically marginalized neighborhoods, to give birth at hospitals in metropolitan Toronto (and Central Ontario for the Asian racial group), and to have low prepregnancy BMI. Participants in the Black and other racial groups were more likely to be multiparous. Those in the Asian and Black racial groups were less likely to have preexisting physical or mental health concerns, to smoke during pregnancy, and to have a midwife as their primary antenatal health care professional; they were more likely to have a physician as a health care professional and to have an infant with a birth weight of 2500 to 3999 g. Participants in the Asian, Black, and other racial groups were less likely to have skin-to-skin contact within the first 2 hours postpartum, and those in the Asian racial group were less likely to initiate breastfeeding within 2 hours.

**Table 1.** Sociodemographic, Health, and Perinatal Characteristics by Quintiles of Socioeconomic Marginalization

Characteristic^a^	No. (%)						
	Total (n = 422 048)	Quintile					Missing (n = 11 092)
		1st (n = 75 216)	2nd (n = 91 942)	3rd (n = 86 881)	4th (n = 75 281)	5th (n = 81 636)	
Sociodemographics							
Maternal age, y							
15-19	3830 (0.9)	267 (0.4)	432 (0.5)	605 (0.7)	775 (1.0)	1480 (1.8)^b^	271 (2.4)
20-24	30 661 (7.3)	2674 (3.6)	4438 (4.8)	5320 (6.1)^b^	6471 (8.6)^b^	10 435 (12.8)^b^	1323 (11.9)
25-29	108 197 (25.6)	15 460 (20.6)	21 606 (23.5)	22 487 (25.9)^b^	21 142 (28.1)^b^	24 338 (29.8)^b^	3164 (28.5)
30-34	171 784 (40.7)	34 555 (45.9)	40 350 (43.9)	36 229 (41.7)	29 257 (38.9)^b^	27 513 (33.7)^b^	3880 (35.0)
35-39	91 818 (21.8)	19 302 (25.7)	21 613 (23.5)	19 060 (21.9)	14 999 (19.9)^b^	14 857 (18.2)^b^	1987 (17.9)
≥40	14 594 (3.5)	2830 (3.8)	3342 (3.6)	3046 (3.5)	2511 (3.3)	2869 (3.5)	356 (3.2)
Missing	804 (0.2)	128 (0.2)	161 (0.2)	134 (0.2)	126 (0.2)	144 (0.2)	111 (1.0)
Maternal race^c^							
Asian	118 082 (28.0)	14 733 (19.6)	25 380 (27.6)^b^	28 026 (32.3)^b^	24 175 (32.1)^b^	23 893 (29.3)^b^	1875 (16.9)
Black	28 876 (6.8)	2011 (2.7)	3303 (3.6)	4659 (5.4)^b^	5547 (7.4)^b^	12 496 (15.3)^b^	860 (7.8)
White	250 463 (59.3)	54 714 (72.7)	58 698 (63.8)^b^	49 764 (57.3)^b^	40 935 (54.4)^b^	39 423 (48.3)^b^	6929 (62.5)
Other^d^	22 122 (5.2)	3488 (4.6)	4108 (4.5)	3975 (4.6)	4140 (5.5)	5064 (6.2)	1347 (12.1)
Missing	2505 (0.6)	270 (0.4)	453 (0.5)	457 (0.5)	484 (0.6)	760 (0.9)	81 (0.7)
Birth hospital region							
Eastern Ontario	59 986 (14.2)	17 724 (23.6)	12 861 (14.0)^b^	9294 (10.7)^b^	8041 (10.7)^b^	8397 (10.3)^b^	3669 (33.1)
Central Ontario	159 154 (37.7)	23 592 (31.4)	42 953 (46.7)^b^	42 512 (48.9)^b^	28 280 (37.6)^b^	19 918 (24.4)^b^	1899 (17.1)
Metro Toronto	118 304 (28.0)	18 305 (24.3)	19 171 (20.9)	19 942 (23.0)	23 253 (30.9)^b^	35 451 (43.4)^b^	2182 (19.7)
Southwestern Ontario	70 247 (16.6)	13 528 (18.0)	14 786 (16.1)	12 411 (14.3)^b^	12 651 (16.8)	14 432 (17.7)	2439 (22.0)
Northern Ontario	14 357 (3.4)	2067 (2.7)	2171 (2.4)	2722 (3.1)	3056 (4.1)	3438 (4.2)	903 (8.1)
Rural residence							
No	373 472 (88.5)	64 796 (86.1)	79 808 (86.8)	76 889 (88.5)	68 674 (91.2)^b^	78 517 (96.2)^b^	4788 (43.2)
Yes	44 072 (10.4)	10 420 (13.9)	12 134 (13.2)	9992 (11.5)	6607 (8.8)^b^	3119 (3.8)^b^	1800 (16.2)
Missing	4504 (1.1)	0 (0)	0 (0)	0 (0)	0 (0)	0 (0)	4504 (40.6)
Multiparity (≥1 previous birth)							
No	344 545 (81.6)	64 167 (85.3)	76 907 (83.6)	71 662 (82.5)	61 520 (81.7)	61 317 (75.1)^b^	8972 (80.9)
Yes	76 305 (18.1)	10 824 (14.4)	14 830 (16.1)	15 059 (17.3)	13 572 (18.0)	20 007 (24.5)^b^	2013 (18.1)
Missing	1198 (0.3)	225 (0.3)	205 (0.2)	160 (0.2)	189 (0.3)	312 (0.4)	107 (1.0)
Maternal health characteristics							
Prepregnancy body mass index^e^							
<18.5	66 089 (15.7)	9590 (12.7)	13 691 (14.9)	14 702 (16.9)^b^	12 921 (17.2)^b^	13 618 (16.7)^b^	1567 (14.1)
18.5-24.9	195 039 (46.2)	39 752 (52.9)	44 698 (48.6)	39 619 (45.6)^b^	32 525 (43.2)^b^	33 392 (40.9)^b^	5053 (45.6)
25.0-29.9	93 213 (22.1)	15 911 (21.2)	19 870 (21.6)	19 068 (21.9)	17 076 (22.7)	18 836 (23.1)	2452 (22.1)
30.0-34.9	40 042 (9.5)	6043 (8.0)	8313 (9.0)	8025 (9.2)	7430 (9.9)	9073 (11.1)^b^	1158 (10.4)
≥35	27 665 (6.6)	3920 (5.2)	5370 (5.8)	5467 (6.3)	5329 (7.1)^b^	6717 (8.2)	862 (7.8)
Any preexisting health condition							
No	334 018 (79.1)	58 265 (77.5)	72 409 (78.8)	69 154 (79.6)	59 975 (79.7)	65 527 (80.3)	8688 (78.2)
Yes	88 030 (20.9)	16 951 (22.5)	19 533 (21.2)	17 727 (20.4)	15 306 (20.3)	16 109 (19.7)	2404 (21.7)
Any mental health concern							
No	347 478 (82.3)	61 061 (81.2)	76 377 (83.1)	72 424 (83.4)	62 042 (82.4)	66 792 (81.8)	8782 (79.2)
Yes	69 204 (16.4)	12 880 (17.1)	14 409 (15.7)	13 446 (15.5)	12 401 (16.5)	13 968 (17.1)	2100 (18.9)
Missing	5366 (1.3)	1275 (1.7)	1156 (1.3)	1011 (1.2)	838 (1.1)	876 (1.1)	210 (1.9)
Smoking at time of birth							
No	397 055 (94.1)	71 374 (94.9)	87 777 (95.5)	82 633 (95.1)	70 555 (93.7)	74 714 (91.5)^b^	10 002 (90.2)
Yes	16 968 (4.0)	1546 (2.1)	2485 (2.7)	2842 (3.3)	3534 (4.7)^b^	5729 (7.0)^b^	832 (7.5)
Missing	8025 (1.9)	2296 (3.1)	1680 (1.8)	1406 (1.6)	1192 (1.6)	1193 (1.5)^b^	258 (2.3)
Drug use during pregnancy							
No	412 113 (97.6)	73 513 (97.7)	90 130 (98.0)	85 216 (98.1)	73 619 (97.8)	79 041 (96.8)	10 594 (95.5)
Yes	1518 (0.4)	158 (0.2)	263 (0.3)	254 (0.3)	276 (0.4)	457 (0.6)	110 (1.0)
Missing	8417 (2.0)	1545 (2.1)	1549 (1.7)	1411 (1.6)	1386 (1.8)	2138 (2.6)	388 (3.5)
Cannabis use during pregnancy							
No	409 135 (96.9)	73 182 (97.3)	89 678 (97.5)	84 763 (97.6)	72 984 (96.9)	78 055 (95.6)	10 473 (94.4)
Yes	6956 (1.6)	713 (0.9)	1014 (1.1)	1087 (1.3)	1410 (1.9)	2369 (2.9)^b^	363 (3.3)
Missing	5957 (1.4)	1321 (1.8)	1250 (1.4)	1031 (1.2)	887 (1.2)	1212 (1.5)	256 (2.3)
Perinatal characteristics and care provision							
Antenatal health care professional							
Physician	353 213 (83.7)	59 870 (79.6)	75 937 (82.6)	72 936 (83.9)^b^	63 888 (84.9)^b^	70 692 (86.6)^b^	9890 (89.2)
Midwife	45 617 (10.8)	10 214 (13.6)	10 658 (11.6)	9350 (10.8)	7533 (10.0)^b^	7180 (8.8)^b^	682 (6.1)
Other	542 (0.1)	78 (0.1)	74 (0.1)	84 (0.1)	109 (0.1)	163 (0.2)	34 (0.3)
Shared care	18 561 (4.4)	3956 (5.3)	4361 (4.7)	3833 (4.4)	3176 (4.2)	2931 (3.6)	304 (2.7)
Missing	4115 (1.0)	1098 (1.5)	912 (1.0)	678 (0.8)	575 (0.8)	670 (0.8)	182 (1.6)
Study year of birth^f^							
Year 1	66 304 (15.7)	11 284 (15.0)	14 277 (15.5)	13 821 (15.9)	11 702 (15.5)	13 200 (16.2)	2020 (18.2)
Year 2	69 685 (16.5)	12 559 (16.7)	14 898 (16.2)	14 423 (16.6)	12 250 (16.3)	13 587 (16.6)	1968 (17.7)
Year3	69 389 (16.4)	12 378 (16.5)	14 903 (16.2)	13 822 (15.9)	12 368 (16.4)	13 646 (16.7)	2312 (20.8)
Year 4	71 432 (16.9)	12 948 (17.2)	15 768 (17.1)	14 760 (17.0)	12 685 (16.9)	13 595 (16.7)	1676 (15.1)
Year 5	72 805 (17.3)	13 025 (17.3)	15 975 (17.4)	15 103 (17.4)	13 180 (17.5)	13 977 (17.1)	1545 (13.9)
Year 6	72 433 (17.2)	13 022 (17.3)	16 121 (17.5)	14 952 (17.2)	13 136 (17.4)	13 631 (16.7)	1571 (14.2)
Mode of delivery							
Vaginal	302 685 (71.7)	54 254 (72.1)	66 436 (72.3)	62 397 (71.8)	53 487 (71.0)	58 236 (71.3)	7875 (71.0)
Cesarean	119 273 (28.3)	20 962 (27.9)	25 506 (27.7)	24 484 (28.2)	21 794 (29.0)	23 400 (28.7)	3127 (28.2)
Missing	90 (0.0)	0 (0)	0 (0)	0 (0)	0 (0)	0 (0)	90 (0.8)
Gestational age at birth, wk							
37-38	119 437 (28.3)	19 603 (26.1)	25 218 (27.4)	24 966 (28.7)	21 902 (29.1)	24 712 (30.3)	3056 (27.6)
39-40	250 872 (59.4)	45 487 (60.5)	55 220 (60.1)	51 504 (59.3)	44 405 (59.0)	47 652 (58.4)	6604 (59.5)
41	50 369 (11.9)	9811 (13.0)	11 214 (12.2)	10 158 (11.7)	8751 (11.6)	9038 (11.1)	1397 (12.6)
≥42	1350 (0.3)	315 (0.4)	290 (0.3)	253 (0.3)	223 (0.3)	234 (0.3)	35 (0.3)
Infant birth weight, g							
2500-3999	380 071 (90.1)	67 031 (89.1)	82 661 (89.9)	78 479 (90.3)	68 161 (90.5)	73 925 (90.6)	9814 (88.5)
4000-4500	36 697 (8.7)	7196 (9.6)	8169 (8.9)	7339 (8.4)	6221 (8.3)	6673 (8.2)	1099 (9.9)
≥4500	5280 (1.3)	989 (1.3)	1112 (1.2)	1063 (1.2)	899 (1.2)	1038 (1.3)	179 (1.6)
Infant sex							
Female	206 894 (49.0)	36 898 (49.1)	45 309 (49.3)	42 393 (48.8)	36 901 (49.0)	39 959 (48.9)	5434 (49.0)
Male	215 016 (50.9)	38 292 (50.9)	46 609 (50.7)	44 465 (51.2)	38 356 (51.0)	41 644 (51.0)	5650 (50.9)
Missing	138 (0)	26 (0)	24 (0)	23 (0)	24 (0)	33 (0)	8 (0.1)
Neonatal intensive care unit admission							
No	399 592 (94.7)	71 361 (94.9)	87 318 (95.0)	82 320 (94.8)	71 035 (94.4)	77 158 (94.5)	10 400 (93.8)
Yes	22 456 (5.3)	3855 (5.1)	4624 (5.0)	4561 (5.2)	4246 (5.6)	4478 (5.5)	692 (6.2)
Intention to breastfeed							
No	3237 (0.8)	356 (0.5)	604 (0.7)	621 (0.7)	597 (0.8)	933 (1.1)	126 (1.1)
Yes	403 495 (95.6)	72 652 (96.6)	88 037 (95.8)	82 645 (95.1)	71 540 (95.0)	78 091 (95.7)	10 530 (94.9)
Unknown/unsure	1936 (0.5)	236 (0.3)	339 (0.4)	396 (0.5)	407 (0.5)	468 (0.6)	90 (0.8)
Missing	13 380 (3.2)	1972 (2.6)	2962 (3.2)	3219 (3.7)	2737 (3.6)	2144 (2.6)	346 (3.1)
Skin-to-skin contact ≥1 h in 1st 2 h after birth							
No	15 663 (3.7)	2578 (3.4)	2975 (3.2)	2951 (3.4)	2959 (3.9)	3669 (4.5)	531 (4.8)
Yes	343 444 (81.4)	63 285 (84.1)	74 138 (80.6)	68 549 (78.9)^b^	60 686 (80.6)	67 444 (82.6)	9342 (84.2)
Missing	62 941 (14.9)	9353 (12.4)	14 829 (16.1)^b^	15 381 (17.7)^b^	11 636 (15.5)	10 523 (12.9)	1219 (11.0)
Breastfeeding initiation within 2 h of birth							
No	9827 (2.3)	1860 (2.5)	2058 (2.2)	1853 (2.1)	1713 (2.3)	2059 (2.5)	284 (2.6)
Yes	300 981 (71.3)	52 791 (70.2)	66 535 (72.4)	63 159 (72.7)	53 268 (71.2)	57 359 (70.3)	7509 (67.7)
Missing	111 240 (26.4)	20 565 (27.3)	23 349 (25.4)	21 869 (25.2)	19 940 (26.5)	22 218 (27.2)	3299 (29.7)
Postpartum breastfeeding support provided							
No	4779 (1.1)	846 (1.1)	877 (1.0)	903 (1.0)	949 (1.3)	1064 (1.3)	140 (1.3)
Yes	395 284 (93.7)	68 837 (91.5)	85 914 (93.4)	82 072 (94.5)^b^	71 042 (94.4)^b^	77 051 (94.4)^b^	10 368 (93.5)
Missing	21 985 (5.2)	5533 (7.4)	5151 (5.6)	3906 (4.5)^b^	3290 (4.4)^b^	3521 (4.3)^b^	584 (5.3)

^a^ All data were derived from individual health record data, except socioeconomic marginalization quintiles were derived from maternal postal codes and 2021 Ontario Marginalization Index–Material Resources;^23^ rural residence was derived from maternal postal codes.

^b^ Standardized difference more than 0.10, comparing participants within each respective quintile to quintile 1 (least marginalized).

^c^ It is unknown whether maternal race data were self-identified or determined by health care professionals.

^d^ Other racial group includes individuals of Indigenous, multiracial, and unknown race.

^e^ Calculated as weight in kilograms divided by height in meters squared.

^f^ Year 1: April 1, 2015-March 31, 2016; year 2: April 1, 2016-March 31, 2017; year 3: April 1, 2017-March 31, 2018; year 4: April 1, 2018-March 31, 2019; year 5: April 1, 2019-March 31, 2020; year 6: April 1, 2020-March 31, 2021.

**Table 2.** Sociodemographic, Health, and Perinatal Characteristics by Maternal Asian, Black, White, or Other Race

Characteristic^a^	No. (%)					
	Total (n = 422 048)^b^	Asian (n = 118 082)	Black (n = 28 876)	White (n = 250 463)	Other (n = 22 122)^c^	Missing (n = 2505)
Sociodemographics						
Maternal age, y						
15-19	3830 (0.9)	186 (0.2)^d^	469 (1.6)	2626 (1.0)	514 (2.3)	35 (1.4)
20-24	30 661 (7.3)	4890 (4.1)^d^	3292 (11.4)^d^	19 685 (7.9)	2536 (11.5)^d^	258 (10.3)
25-29	108 197 (25.6)	28 952 (24.5)	7520 (26.0)	65 304 (26.1)	5686 (25.7)	735 (29.3)
30-34	171 784 (40.7)	50 526 (42.8)	9515 (33.0)^d^	103 056 (41.1)	7814 (35.3)^d^	873 (34.9)
35-39	91 818 (21.8)	28 412 (24.1)	6408 (22.2)	51 792 (20.7)	4698 (21.2)	508 (20.3)
≥40	14 954 (3.5)	4884 (4.1)	1628 (5.6)^d^	7520 (3.0)	830 (3.8)	92 (3.7)
Missing	804 (0.2)	232 (0.2)	44 (0.2)	480 (0.2)	44 (0.2)	NR
Socioeconomic marginalization						
Quintile 1 (least marginalized)	75 216 (17.8)	14 733 (12.5)^d^	2011 (7.0)^d^	54 714 (21.8)	3488 (15.8)^d^	270 (10.8)
Quintile 2	91 942 (21.8)	25 380 (21.5)	3303 (11.4)^d^	58 698 (23.4)	4108 (18.6)^d^	453 (18.1)
Quintile 3	86 881 (20.6)	28 026 (23.7)	4659 (16.1)	49 764 (19.9)	3975 (18.0)	457 (18.2)
Quintile 4	75 281 (17.8)	24 175 (20.5)^d^	5547 (19.2)	40 935 (16.3)	4140 (18.7)	484 (19.3)
Quintile 5 (most marginalized)	81 636 (19.3)	23 893 (20.2)^d^	12 496 (43.3)^d^	39 423 (15.7)	5064 (22.9)^d^	760 (30.3)
Missing	11 092 (2.6)	1875 (1.6)	860 (3.0)	6929 (2.8)	1347 (6.1)^d^	81 (3.2)
Birth hospital region						
Eastern Ontario	59 986 (14.2)	4853 (4.1)^d^	2885 (10.0)^d^	48 432 (19.3)	3705 (16.7)	111 (4.4)
Central Ontario	159 154 (37.7)	53 346 (45.2)^d^	9095 (31.5)	88 865 (35.5)	7208 (32.6)	640 (25.5)
Metro Toronto	118 304 (28.0)	51 774 (43.8)^d^	14 462 (50.1)^d^	43 999 (17.6)	6582 (29.8)^d^	1487 (59.4)
Southwestern Ontario	70 247 (16.6)	7614 (6.4)^d^	2280 (7.9)^d^	57 148 (22.8)	3069 (13.9)^d^	136 (5.4)
Northern Ontario	14 357 (3.4)	495 (0.4)^d^	154 (0.5)^d^	12 019 (4.8)	1558 (7.0)	131 (5.2)
Rural residence						
No	373 472 (88.5)	115 854 (98.1)^d^	28 196 (97.6)^d^	207 563 (82.9)	19 561 (88.4)^d^	2298 (91.7)
Yes	44 072 (10.4)	1154 (1.0)^d^	326 (1.1)^d^	40 201 (16.1)	2237 (10.1)^d^	154 (6.1)
Missing	4504 (1.1)	1074 (0.9)	354 (1.2)	2699 (1.1)	324 (1.5)	53 (2.1)
Multiparity (≥1 previous birth)						
No	344 545 (81.6)	96 886 (82.0)	19 345 (67.3)^d^	208 981 (83.4)	17 349 (78.4)^d^	1894 (75.6)
Yes	76 305 (18.1)	20 724 (17.6)	9336 (32.3)^d^	40 940 (16.3)	4698 (21.2)^d^	607 (24.2)
Missing	1198 (0.3)	472 (0.4)	105 (0.4)	542 (0.2)	75 (0.3)	NR
Maternal health characteristics						
Prepregnancy body mass index^e^						
<18.5	66 089 (15.7)	27 678 (23.4)^d^	6148 (21.3)^d^	28 506 (11.4)	3350 (15.1)^d^	407 (16.2)
18.5-24.9	195 039 (46.2)	58 981 (49.9)	9648 (33.4)^d^	115 818 (46.2)	9446 (42.7)	1146 (45.7)
25.0-29.9	93 213 (22.1)	21 975 (18.6)^d^	7213 (25.0)	58 219 (23.2)	5216 (23.6)	590 (23.6)
30.0-34.9	40 042 (9.5)	6884 (5.8)^d^	3551 (12.3)	26 915 (10.7)	2453 (11.1)	239 (9.5)
>35	27 665 (6.6)	2564 (2.2)^d^	2316 (8.0)	21 005 (8.4)	1657 (7.5)	123 (4.9)
Any preexisting health condition						
No	334 018 (79.1)	97 766 (82.8)^d^	23 466 (81.3)^d^	193 283 (77.2)	17 358 (78.5)	2145 (85.6)
Yes	88 030 (20.9)	20 316 (17.2)^d^	5410 (18.7)^d^	57 180 (22.8)	4764 (21.5)	360 (14.4)
Any mental health concern						
No	347 478 (82.3)	110 335 (93.4)^d^	25 765 (89.2)^d^	191 559 (76.5)	17 564 (79.4)	2255 (90.0)
Yes	69 204 (16.4)	6107 (5.2)^d^	2688 (9.3)^d^	56 010 (22.4)	4166 (18.8)	233 (9.3)
Missing	5366 (1.3)	1640 (1.4)	423 (1.5)	2894 (1.2)	392 (1.8)	17 (0.7)
Smoking at time of birth						
No	397 055 (94.1)	115 274 (97.6)^d^	27 700 (95.9)^d^	231 487 (92.4)	20 203 (91.3)	2391 (95.4)
Yes	16 968 (4.0)	559 (0.5)^d^	547 (1.9)^d^	14 447 (5.8)	1331 (6.0)	84 (3.4)
Missing	8025 (1.9)	2249 (1.9)	629 (2.2)	4529 (1.8)	588 (2.7)	30 (1.2)
Drug use during pregnancy						
No	412 113 (97.6)	116 282 (98.5)	28 084 (97.3)	244 010 (97.4)	21 288 (96.2)	2449 (97.8)
Yes	1518 (0.4)	150 (0.1)	73 (0.3)	1071 (0.4)	207 (0.9)	17 (0.7)
Missing	8417 (2.0)	1650 (1.4)	719 (2.5)	5382 (2.1)	627 (2.8)	39 (1.6)
Cannabis use during pregnancy						
No	409 135 (96.9)	116 148 (98.4)^d^	27 823 (96.4)	241 692 (96.5)	21 027 (95.1)	2445 (97.6)
Yes	6956 (1.6)	410 (0.3)^d^	581 (2.0)	5257 (2.1)	683 (3.1)	25 (1.0)
Missing	5957 (1.4)	1524 (1.3)	472 (1.6)	3514 (1.4)	412 (1.9)	35 (1.4)
Perinatal characteristics and care provision						
Antenatal health care professional						
Physician	353 213 (83.7)	108 109 (91.6)^d^	25 324 (87.7)^d^	199 245 (79.6)	18 047 (81.6)	2308 (92.1)
Midwife	45 617 (10.8)	6081 (5.1)^d^	2221 (7.7)^d^	34 585 (13.8)	2611 (11.8)	119 (4.8)
Other	542 (0.1)	111 (0.1)	32 (0.1)	344 (0.1)	54 (0.2)	NR
Shared care	18 561 (4.4)	2532 (2.1)^d^	984 (3.4)^d^	13 882 (5.5)	1100 (5.0)	63 (2.5)
Missing	4115 (1.0)	1249 (1.1)	315 (1.1)	2227 (0.9)	310 (1.4)	14 (0.6)
Study year of birth^f^						
Year 1	66 304 (15.7)	17 707 (15.0)	4367 (15.1)	40 377 (16.1)	2915 (13.2)	938 (37.4)
Year 2	69 685 (16.5)	19 194 (16.3)	4600 (15.9)	41 642 (16.6)	3509 (15.9)	740 (29.5)
Year 3	69 389 (16.4)	18 669 (15.8)	4630 (16.0)	41 610 (16.6)	4036 (18.2)	444 (17.7)
Year 4	71 432 (16.9)	19 897 (16.9)	4820 (16.7)	42 554 (17.0)	4042 (18.3)	119 (4.8)
Year 5	72 805 (17.3)	20 936 (17.7)	5140 (17.8)	42 781 (17.1)	3831 (17.3)	117 (4.7)
Year 6	72 433 (17.2)	21 679 (18.4)	5319 (18.4)	41 499 (16.6)	3789 (17.1)	147 (5.9)
Mode of delivery						
Vaginal	302 685 (71.7)	84 487 (71.5)	19 657 (68.1)	181 295 (72.4)	15 392 (69.6)	1854 (74.0)
Cesarean	119 273 (28.3)	33 556 (28.4)	9213 (31.9)	69 125 (27.6)	6728 (30.4)	651 (26.0)
Missing	90 (0.0)	39 (0)	6 (0)	43 (0)	NR	0 (0)
Gestational age at birth, wk						
37-38	119 457 (28.3)	38 546 (32.6)^d^	8510 (29.5)	65 035 (26.0)	6676 (30.2)	690 (27.5)
39-40	250 872 (59.4)	69 858 (59.2)	16 982 (58.8)	149 573 (59.7)	12 942 (58.5)	1517 (60.6)
41	50 369 (11.9)	9535 (8.1)^d^	3291 (11.4)	34 838 (13.9)	2416 (10.9)	289 (11.5)
≥42	1350 (0.3)	143 (0.1)	93 (0.3)	1017 (0.4)	88 (0.4)	9 (0.4)
Infant birth weight, g						
2500-3999	380 071 (90.1)	112 481 (95.3)^d^	26 441 (91.6)^d^	219 128 (87.5)	19 706 (89.1)	2315 (92.4)
4000-4500	36 697 (8.7)	5067 (4.3)^d^	2133 (7.4)^d^	27 252 (10.9)	2074 (9.4)	171 (6.8)
≥4500	5280 (1.3)	534 (0.5)^d^	302 (1.0)	4083 (1.6)	342 (1.5)	19 (0.8)
Infant sex						
Female	206 894 (49.0)	57 875 (49.0)	14 255 (49.4)	122 687 (49.0)	10 857 (49.1)	1220 (48.7)
Male	214 016 (50.9)	60 180 (51.0)	14 613 (50.6)	127 679 (51.0)	11 259 (50.9)	1285 (51.3)
Missing	138 (0.0)	27 (0)	8 (0)	97 (0)	6 (0)	0 (0)
Neonatal intensive care unit admission						
No	399 592 (94.7)	112 225 (95.0)	27 273 (94.4)	236 876 (94.6)	20 866 (94.3)	2352 (93.9)
Yes	22 456 (5.3)	5857 (5.0)	1603 (5.6)	13 587 (5.4)	1256 (5.7)	153 (6.1)
Intention to breastfeed						
No	3237 (0.8)	877 (0.7)	189 (0.7)	1968 (0.8)	184 (0.8)	19 (0.8)
Yes	403 495 (95.6)	111 493 (94.4)	27 398 (94.9)	241 245 (96.3)	20 960 (94.7)	2399 (95.8)
Unknown/unsure	1936 (0.5)	309 (0.3)	116 (0.4)	1363 (0.5)	135 (0.6)	13 (0.5)
Missing	13 380 (3.2)	5403 (4.6)^d^	1173 (4.1)	5887 (2.4)	843 (3.8)	74 (3.0)
Skin-to-skin contact ≥1 h in 1st 2 h after birth						
No	15 663 (3.7)	4165 (3.5)	1316 (4.6)	9183 (3.7)	865 (3.9)	134 (5.3)
Yes	343 444 (81.4)	88 406 (74.9)^d^	22 358 (77.4)^d^	212 899 (85.0)	17 770 (80.3)^d^	2011 (80.3)
Missing	62 941 (14.9)	25 511 (21.6)^d^	5202 (18.0)^d^	28 381 (11.3)	3487 (15.8)^d^	360 (14.4)
Breastfeeding initiation within 2 h of birth						
No	9827 (2.3)	2455 (2.1)	743 (2.6)	6054 (2.4)	559 (2.5)	16 (0.6)
Yes	300 981 (71.3)	80 788 (68.4)^d^	20 189 (69.9)	182 924 (73.0)	15 473 (69.9)	1607 (64.2)
Missing	111 240 (26.4)	34 839 (29.5)^d^	7944 (27.5)	61 485 (24.5)	6090 (27.5)	882 (35.2)
Postpartum breastfeeding support provided						
No	4779 (1.1)	1362 (1.2)	367 (1.3)	2691 (1.1)	338 (1.5)	21 (0.8)
Yes	395 284 (93.7)	110 911 (93.9)	27 260 (94.4)	234 253 (93.5)	20 451 (92.4)	2409 (96.2)
Missing	21 985 (5.2)	5809 (4.9)	1249 (4.3)	13 519 (5.4)	1333 (6.0)	75 (3.0)

Abbreviation: NR, data not reported due to small cell count, per BORN privacy policies.

^a^ All data from individual health record data, except socioeconomic marginalization quintiles were derived from maternal postal codes and 2021 Ontario Marginalization Index–Material Resources;^23^ rural residence was derived from maternal postal codes.

^b^ It is unknown whether maternal race data were self-identified or determined by health care professionals.

^c^ Other racial group includes individuals of Indigenous, multiracial, and unknown race.

^d^ Standardized difference more than 0.10, comparing participants within each respective racial group to the White racial group.

^e^ Calculated as weight in kilograms divided by height in meters squared.

^f^ Year 1: April 1, 2015-March 31, 2016; year 2: April 1, 2016-March 31, 2017; year 3: April 1, 2017-March 31, 2018; year 4: April 1, 2018-March 31, 2019; year 5: April 1, 2019-March 31, 2020; year 6: April 1, 2020-March 31, 2021.

Overall, 27.3% of infants received nonmedically indicated hospital formula supplementation, with prevalence increasing from 23.4% in year 1 to 25.7%, 27.4%, 28.0%, 27.0%, and 31.8% across years 2 through 6 (Figure). A further 3.9% of infants were supplemented for medical reasons across all study years. Reasons for supplementation were missing for 6.4% of participants (17.0% of those supplemented) with consistent distribution across exposure groups (eTable 2 in Supplement 1).

**Figure.**
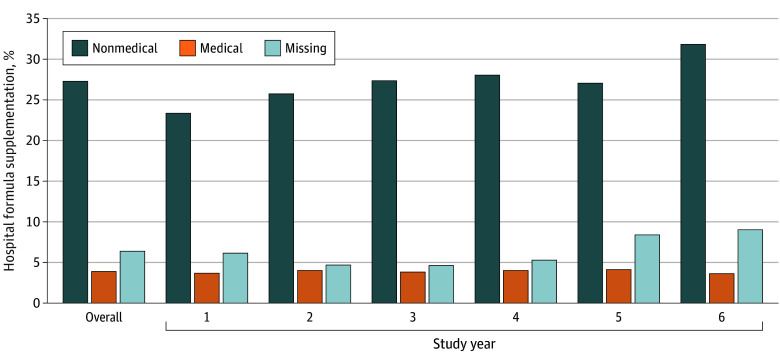
Prevalence of Hospital Formula Supplementation According to Nonmedical or Medical Indication (n = 422 048) Year 1: April 1, 2015, to March 31, 2016; year 2: April 1, 2016, to March 31, 2017; year 3: April 1, 2017, to March 31, 2018; year 4: April 1, 2018, to March 31, 2019; year 5: April 1, 2019, to March 31, 2020; year 6: April 1, 2020, to March 31, 2021.

A gradient of increased risk of non-medically indicated formula supplementation was found across quintiles 2 through 5 compared with quintile 1 (Table 3). This pattern was consistent across models, with adjusted model 2 showing quintile 5 participants at 1.7-times greater risk (aRR, 1.68; 95% CI, 1.64-1.72).

**Table 3.** Associations Between Maternal Socioeconomic Marginalization Quintiles and Nonmedically Indicated Formula Supplementation (n = 384 656)

Quintile	Frequency, No. (%)	RR (95% CI)		
		Unadjusted	Model, adjusted^a^	
			1	2
1	14 983 (3.9)	1 [Reference]	1 [Reference]	1 [Reference]
2	22 209 (5.8)	1.29 (1.26-1.32)	1.18 (1.15-1.21)	1.17 (1.14-1.20)
3	23 811 (6.2)	1.55 (1.52-1.59)	1.33 (1.30-1.37)	1.31 (1.28-1.35)
4	22 674 (5.9)	1.78 (1.74-1.83)	1.51 (1.47-1.55)	1.47 (1.43-1.50)
5	28 318 (7.4)	2.22 (2.17-2.27)	1.77 (1.73-1.82)	1.68 (1.64-1.72)

Abbreviation: RR, relative risk.

^a^ Model 1 adjusted for maternal sociodemographics (age, race, rural residence, birth hospital region, parity). Model 2 adjusted for model 1 covariates and maternal health characteristics (prepregnancy body mass index, preexisting health conditions, mental health concerns, prenatal smoking, prenatal substance use, prenatal cannabis use). Akaike information criterion values: model 1, 458 907; model 2, 440 056.

Maternal race was significantly associated with nonmedically indicated formula supplementation in unadjusted analysis and the association remained robust in the adjusted models (Table 4). Compared with the White racial group, adjusted model 2 showed a 2.7-fold risk for participants in the Asian group (aRR, 2.69; 95% CI, 2.64-2.74) and more than 2-fold risk for those in the Black group (aRR, 2.07; 95% CI, 2.01-2.13). Participants in the other racial group were 1.4 times more likely to receive nonmedically indicated formula supplementation (aRR, 1.43; 95% CI, 1.39-1.48).

**Table 4.** Associations Between Maternal Race and Nonmedically Indicated Formula Supplementation (n = 316 214)

Race	Frequency, No. (%)	RR (95% CI)		
		Unadjusted	Model, adjusted^a^	
			1	2
Asian	47 056 (14.9)	2.89 (2.85-2.94)	2.55 (2.51-2.59)	2.69 (2.64-2.74)
Black	11 146 (35.2)	2.68 (2.61-2.75)	2.09 (2.03-2.15)	2.07 (2.01-2.13)
White	50 096 (15.8)	1 [Reference)	1 [Reference]	1 [Reference]
Other	6101 (1.9)	1.58 (1.53-1.63)	1.42 (1.38-1.47)	1.43 (1.39-1.48)

Abbreviation: RR, relative risk.

^a^ Model 1 adjusted for maternal sociodemographics (age, 2021 Ontario Marginalization Index quintile, rural residence, birth hospital region, parity). Model 2 adjusted for model 1 covariates and maternal health characteristics (prepregnancy body mass index, preexisting health conditions, mental health concerns, prenatal smoking, prenatal substance use, prenatal cannabis use). Akaike information criterion values: model 1, 458 907; model 2, 440 056.

The greatest increase in the prevalence of nonmedically indicated formula supplementation occurred between year 5 (27.0%) and year 6 (31.8%), the first year of the COVID-19 pandemic (Figure). Subgroup analyses showed consistency between years 1 through 5 and year 6 in the increased risk of nonmedically indicated supplementation for socioeconomic quintiles 2 through 5 compared with quintile 1, and for the Asian, Black, and other racial groups compared with the White group (eTables 3 and 4 in Supplement 1).

Results of the exploratory analysis, which additionally adjusted for perinatal characteristics, were consistent with model 2 results (eTables 5 and 6 in Supplement 1).

## Discussion

This population-based analysis showed a significant association between maternal socioeconomic status and race and nonmedically indicated hospital formula supplementation of term-born breastfed newborns. We found a gradient of increased risk across quintiles of increasing socioeconomic marginalization. For maternal race, the greatest risks were found among the Asian (aRR, 2.69) and Black (aRR, 2.07) groups compared with the White group. These disparities were consistent before and during the first year of the COVID-19 pandemic. To our knowledge, this study contributes the largest population-level analysis of disparities in hospital supplementation, and uniquely focuses on nonmedically indicated supplementation in the context of a publicly-funded health care system. Our findings point to biases in care practices which increase the risk of early breastfeeding cessation among socioeconomically marginalized and racialized families.

We identified 2 prior population-based analyses of disparities in hospital formula supplementation. Nguyen et al^26^ analyzed birth certificate records for all singleton, term-born breastfed infants born in 2014 in 126 New York hospitals (n = 160 911); adjusted odds ratios (aORs) were calculated separately for hospitals offering 4 different levels of care. Supplementation prevalence increased in a gradient with decreasing maternal education level (aOR for ≤ grade 12, 2.01-2.95) and was nearly doubled (61% vs 33%) for the Asian (aOR, 1.85-2.74) and African American racial groups (aOR, 1.54-2.05) compared with the White group. A retrospective cohort study^27^ using medical record data for term births from 2010 through 2013 at 5 hospitals in Australia (n = 24 713) found that maternal birth in an Asian country predicted in-hospital supplementation of infants whose mothers intended to exclusively breastfeed (aRR, 2.07); a gradient of supplementation risk across quartiles of decreasing socioeconomic was also observed (aRR, 1.30 for lowest vs highest quartile). Our findings align with these studies, despite notable differences in setting and timeframe, suggesting that hospital supplementation of breastfed infants from socioeconomically marginalized and racialized families is a widespread and persistent health equity issue. This is consistent with growing evidence of systemic barriers and embedded biases within health care delivery as contributors to disparities in perinatal care and outcomes.^28,29,30^

Several prior nonpopulation-based studies also show evidence of disparities in newborn supplementation practices. Analysis of 2009 through 2015 National Immunization Survey data from the US found higher supplementation prevalence in the first 2 days postpartum with maternal Asian or Black racial identity compared with White, but focused on changes over time rather than magnitude of disparities, and supplementation may have occurred outside the hospital.^19^ Analysis of infant feeding data from a community-based cohort (n = 1636) found that disparities in breastfeeding duration between White and Black participants were mediated by hospital formula supplementation, but findings cannot be generalized to the wider American population.^31^ In a longitudinal cohort study of infants born from 2009 through 2014 in 4 Canadian cities (n = 3195), hospital formula supplementation was associated with lower maternal education and single parenthood, but an adjusted analysis was not performed.^17^ Exclusive breastfeeding in hospital predicted longer breastfeeding duration, with greater effect among mothers without postsecondary education.^17^ A cohort study in the province of Newfoundland and Labrador (n = 451) found significantly higher rates of hospital formula supplementation and shorter breastfeeding duration among participants from low-income households compared with more privileged households; early postpartum experiences were a key determinant of early breastfeeding cessation in the low-income group.^18^ Our findings confirm and strengthen this evidence base through population-level analysis, adjusted for multiple covariates and focused on nonmedically indicated supplementation. We built a cohort of term-born breastfed infants to examine supplementation disparities without confounding from differences in breastfeeding initiation, preterm birth or medical fragility.

Our data show a high overall prevalence of formula supplementation (38%), with an increase from 33% to 45% over the study period; at least 73% was not medically indicated. This translates to an in-hospital exclusive breastfeeding rate of 62%, well below the targets set globally (80%) and nationally (75%).^8,32^ In contrast, US National Immunization Survey data for births in 2019 found that 19% of breastfed newborns received supplementation within the first 2 days postpartum.^33^ These data were collected from parents at 19 through 35 months postpartum and may be affected by recall bias, but the lower supplementation rate may also reflect the spread of the BFI in the US, with 29% of births in BFI-certified hospitals.^33^ Uptake of the BFI lags significantly in Canada, with fewer than 10% of eligible hospitals participating.^34^ The 2019 Ontario BFI scorecard reported lower prevalence of nonmedically indicated supplementation among BFI-designated hospitals (19% vs 30%).^16^ Further research examining disparities in other BFI practices and associations with later breastfeeding outcomes among the diverse Canadian population is needed. Strengthened adherence to BFI principles benefits all infants and has been shown to reduce racial and socioeconomic breastfeeding disparities in the US.^20,35,36,37^

### Limitations

This study has several limitations. First, the exposure variables were sourced through prenatal screening records submitted to BORN. Uptake of prenatal screening is approximately 70% among singleton term births in Ontario but varies by region; lower uptake is associated with living in a rural area, receiving first-trimester care from a family physician or midwife vs an obstetrician, and being in a lower income quintile.^24^ Comparison of our cohort with births ineligible due to missing prenatal screening data showed consistency with these patterns (eTable 1 in Supplement 1); this potential selection bias may limit generalizability of findings to the Ontario population.

In addition, the broad racial groupings do not capture the diversity within each group, and it is unknown whether maternal race data were self-identified or health care professional-determined. Socioeconomic status was derived from neighborhood-level data, which may not accurately represent individual participants despite using the multidimensional ON-Marg Index to enhance robustness of this variable. BORN is working to improve the quality of data on social determinants of health; we recommend updating our analyses once that process is complete. Lastly, despite including multiple covariates, there may be additional unmeasured confounders.

## Conclusions

This population-based analysis found high and increasing prevalence of nonmedically indicated formula supplementation of term-born breastfed newborns in Ontario hospitals. The risk of nonmedically indicated supplementation increased in a gradient across quintiles of increasing socioeconomic marginalization, and was significantly higher among the Asian, Black, and other racial groups compared with the White group. Our findings suggest that breastfeeding among socioeconomically marginalized and racialized families is being undermined by hospital formula provision, compounding health inequities. To promote health equity and optimize breastfeeding outcomes for all families, there is an urgent need to address biases in hospital supplementation practices and improve adherence to evidence-based breastfeeding support guidelines.

## Notes

Supplement 1. eFigure. Derivation of the study cohort eTable 1. Comparison of cohort participants with births ineligible due to missing prenatal screening data eTable 2. Participant characteristics by infant formula supplementation status eTable 3. Sub-group analysis of associations between maternal socio-economic status and non-medically indicated formula supplementation, comparing births in study years prior to the COVID-19 pandemic (Y1-Y5) and births in the first year of the pandemic (Y6) eTable 4. Sub-group analysis of associations between maternal race and non-medically indicated formula supplementation, comparing births in study years prior to the COVID-19 pandemic (Y1-Y5) and births in the first year of the pandemic (Y6) eTable 5. Associations between maternal socio-economic marginalization quintiles and non-medically indicated formula supplementation, adjusted for maternal socio-demographics, maternal health characteristics and perinatal characteristics eTable 6. Associations between maternal race and non-medically indicated formula supplementation, adjusted for maternal socio-demographics, maternal health characteristics and perinatal characteristics

Supplement 2. Data sharing statement
