# Supplementary material for: Hospital Formula Supplementation Postbreastfeeding Initiation, Neighborhood Economy, and Race
Source: JAMA Pediatr. 2025 Dec 29;180(3):263–74. doi: 10.1001/jamapediatrics.2025.5379 (PMC12750333; doi:10.1001/jamapediatrics.2025.5379)
Supplement: Supplement 1. — eFigure. Derivation of the study cohort eTable 1. Comparison of cohort participants with births ineligible due to missing prenatal screening data eTable 2. Participant characteristics by infant formula supplementation status eTable 3. Sub-group analysis of associations between maternal socio-economic status and non-medically indicated formula supplementation, comparing births in study years prior to the COVID-19 pandemic (Y1-Y5) and births in the first year of the pandemic (Y6) eTable 4. Sub-group analysis of associations between maternal race and non-medically indicated formula supplementation, comparing births in study years prior to the COVID-19 pandemic (Y1-Y5) and births in the first year of the pandemic (Y6) eTable 5. Associations between maternal socio-economic marginalization quintiles and non-medically indicated formula supplementation, adjusted for maternal socio-demographics, maternal health characteristics and perinatal characteristics eTable 6. Associations between maternal race and non-medically indicated formula supplementation, adjusted for maternal socio-demographics, maternal health characteristics and perinatal characteristics [file jamapediatr-e255379-s001.pdf]

Supplemental Online Content

Mildon A, Alton GD, Baxter JB, et al. Hospital formula supplementation postbreastfeeding initiation, neighborhood economy, and race. *JAMA Pediatr*. Published online December 29, 2025. doi:10.1001/jamapediatrics.2025.5379

eFigure. Derivation of the study cohort
eTable 1. Comparison of cohort participants with births ineligible due to missing prenatal screening data
eTable 2. Participant characteristics by infant formula supplementation status
eTable 3. Sub-group analysis of associations between maternal socio-economic status and non-medically indicated formula supplementation, comparing births in study years prior to the COVID-19 pandemic (Y1-Y5) and births in the first year of the pandemic (Y6**)**
eTable 4. Sub-group analysis of associations between maternal race and non-medically indicated formula supplementation, comparing births in study years prior to the COVID-19 pandemic (Y1-Y5) and births in the first year of the pandemic (Y6)
eTable 5. Associations between maternal socio-economic marginalization quintiles and non-medically indicated formula supplementation, adjusted for maternal socio-demographics, maternal health characteristics and perinatal characteristics
eTable 6. Associations between maternal race and non-medically indicated formula supplementation, adjusted for maternal socio-demographics, maternal health characteristics and perinatal characteristics

This supplemental material has been provided by the authors to give readers additional information about their work.

© 2025 Mildon A et al. *JAMA Pediatr*.

eFigure 1. Derivation of the study cohort

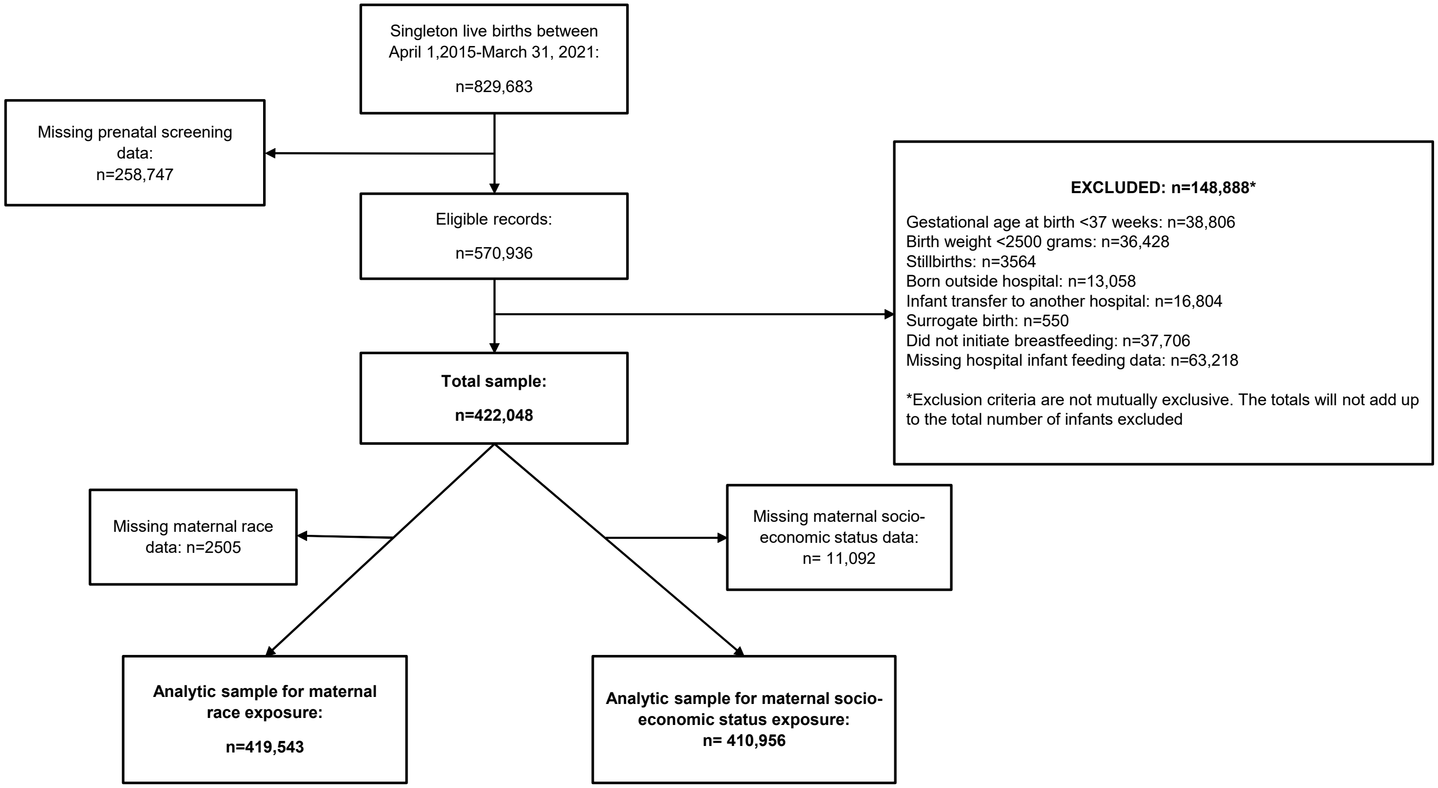

© 2025 Mildon A et al. *JAMA Pediatr*.

eTable 1. Comparison of cohort participants with births ineligible due to missing prenatal screening data

Characteristic	Included in cohort, n (%) (n=422,048)	Ineligible for cohort, n (%) (n=258,747)
Maternal age, y		
15-19	3830 (0.9)	7208 (2.8)
20-24	30,661 (7.3)	36,064 (13.9)
25-29	108,197 (25.6)	74,367 (28.7)
30-34	171,784 (40.7)	82,935 (32.1)
35-39	91,818 (21.8)	42,223 (16.3)
≥40	14,954 (3.5)	15,479 (6.0)
Missing	804 (0.2)	471 (0.2)
Birth hospital region		
Eastern Ontario	59,986 (14.2)	43,668 (16.9)
Central Ontario	159,154 (37.7)	72,660 (28.1)
Metro Toronto	118,304 (28.0)	43,229 (16.7)
Southwestern Ontario	70,247 (16.6)	61,466 (23.8)
Northern Ontario	14,357 (3.4)	24,402 (9.4)
Missing	0 (0.0)	13,322 (5.1)
Rural residence		
No	373,472 (88.5)	204,371 (79.0)
Yes	44,072 (10.4)	49,831 (19.3)
Missing	4504 (1.1)	4545 (1.8)
Multiparity (≥1 previous birth)		
No	344,545 (81.6)	179,191 (69.3)
Yes	76,305 (18.1)	78,423 (30.3)
Missing	1198 (0.3)	1133 (0.4)
Prepregnancy Body Mass Index (kg/m^2)		
<18.5	66,089 (15.7)	45,775 (17.7)
18.5-24.9	195,039 (46.2)	111,812 (43.2)
25.0-29.9	93,213 (22.1)	55,707 (21.5)
30.0-34.9	40,042 (9.5)	25,937 (10.0)
≥35	27,665 (6.6)	19,516 (7.5)
Any pre-existing health condition		
No	334,018 (79.1)	207,188 (80.1)
Yes	88,030 (20.9)	51,559 (19.9)
Any mental health concern		
No	347,478 (82.3)	195,731 (75.6)
Yes	69,204 (16.4)	53,134 (20.5)
Missing	5366 (1.3)	9882 (3.8)
Smoking at time of birth		
No	397,055 (94.1)	224,402 (86.7)
Yes	16,968 (4.0)	27,109 (10.5)
Missing	8025 (1.9)	7236 (2.8)
Drug use during pregnancy		
No	412,113 (97.6)	243,516 (94.1)
Yes	1518 (0.4)	5078 (2.0)
Missing	8417 (2.0)	10,153 (3.9)
Cannabis use during pregnancy		
No	409,135 (96.9)	240,819 (93.1)
Yes	6956 (1.6)	9652 (3.7)
Missing	5957 (1.4)	8276 (3.2)

© 2025 Mildon A et al. *JAMA Pediatr*.

eTable 1. Comparison of cohort participants with births ineligible due to missing prenatal screening data (continued)

Characteristic	Included in cohort, n (%) (n=422,048)	Ineligible for cohort, n (%) (n=258,747)
Antenatal care provider		
Doctor	353,213 (83.7)	185,548 (71.7)
Midwife	45,617 (10.8)	46,273 (17.9)
Other	542 (0.1)	903 (0.3)
Shared care	18,561 (4.4)	14,719 (5.7)
Study year of birth		
Y1 (April 1, 2015-March 31, 2016)	66,304 (15.7)	46,173 (17.8)
Y2 (April 1, 2016-March 31, 2017)	69,685 (16.5)	45,198 (17.5)
Y3 (April 1, 2017-March 31, 2018)	69,389 (16.4)	44,869 (17.3)
Y4 (April 1, 2018-March 31, 2019)	71,432 (16.9)	42,764 (16.5)
Y5 (April 1, 2019-March 31, 2020)	72,805 (17.3)	41,556 (16.1)
Y6 (April 1, 2020-March 31, 2021)	72,433 (17.2)	38,187 (14.8)
Mode of delivery		
Vaginal	302,685 (71.7)	191,708 (74.1)
C-section	119,273 (28.3)	66,976 (25.9)
Missing	90 (0.0)	63 (0.0)
Gestational age at birth, weeks		
37-38	119,457 (28.3)	68,937 (26.6)
39-40	250,872 (59.4)	140,574 (54.3)
41	50,369 (11.9)	29,741 (11.5)
≥42	1350 (0.3)	1522 (0.6)
Missing	0 (0.0)	17,973 (6.9)
Infant birth weight (grams)		
2500-3999	380,071 (90.1)	213,262 (82.4)
4000-4500	36,697 (8.7)	24,562 (9.5)
>4500	5280 (1.3)	4200 (1.6)
Missing	0 (0.0)	16,723 (6.5)
Infant sex		
Female	206,894 (49.0)	125,245 (48.4)
Male	214,016 (50.9)	133,276 (51.5)
Missing	138 (0.0)	226 (0.0)
Neonatal Intensive Care Unit admission		
No	399,592 (94.7)	225,923 (87.3)
Yes	22,456 (5.3)	32,824 (12.7)
Intention to breastfeed		
No	3237 (0.8)	18,137 (7.0)
Yes	403,495 (95.6)	224,508 (86.8)
Unknown/unsure	1936 (0.5)	2930 (1.1)
Missing	13,380 (3.2)	13,172 (5.1)
Skin-to-skin contact ≥ 1 hour		
No	15,663 (3.7)	21,783 (8.4)
Yes	343,444 (81.4)	197,812 (76.4)
Missing	62,941 (14.9)	39,152 (15.1)
Breastfeeding initiation within 2 hrs		
No	9827 (2.3)	11,943 (4.6)
Yes	300,981 (71.3)	160,949 (62.2)
Missing	111,240 (26.4)	85,855 (33.2)
Postpartum breastfeeding support		
No	4779 (1.1)	24,418 (9.4)
Yes	395,284 (93.7)	212,709 (82.2)
Missing	21,985 (5.2)	21,620 (8.4)

© 2025 Mildon A et al. *JAMA Pediatr*.

eTable 2. Participant characteristics by infant formula supplementation status

Characteristic	TOTAL (n=422,048)	Non-medically indicated supplementation (n=115,184)	Medically indicated supplementation (n=16,371)	Missing reason for supplementation (n=26,971)	No supplementation (n=263,522)
**Exposure Variables, No. (%)**					
Socio-economic marginalization					
Quintile 1	75,216 (17.8)	14,997 (13.0)	2917 (17.8)	4224 (15.7)	53,078 (20.1)
Quintile 2	91,942 (21.8)	22,242 (19.3)	3611 (22.1)	5437 (20.2)	60,652 (23.0)
Quintile 3	86,881 (20.6)	23,820 (20.7)	3575 (21.8)	5734 (21.3)	53,752 (20.4)
Quintile 4	75,281 (17.8)	22,714 (19.7)	2862 (17.5)	5235 (19.4)	44,470 (16.9)
Quintile 5	81,636 (19.3)	28,324 (24.6)	3061 (18.7)	5696 (21.1)	44,555 (16.9)
Missing	11,092 (2.6)	3087 (2.7)	345 (2.1)	645 (2.4)	7015 (2.7)
Maternal race					
Asian	118,082 (28.0)	47,056 (40.9)	5309 (32.4)	10,178 (37.7)	55,539 (21.1)
Black	28,876 (6.8)	11,146 (9.7)	1058 (6.5)	2160 (8.0)	14,512 (5.5)
White	250,463 (59.3)	50,096 (43.5)	8991 (54.9)	12,916 (47.9)	178,460 (67.7)
Other[a]	22,122 (5.2)	6101 (5.3)	933 (5.7)	1565 (5.8)	13,523 (5.1)
Missing	2505 (0.6)	785 (0.7)	80 (0.5)	152 (0.6)	1488 (0.6)
**Socio-demographics, No. (%)**					
Maternal age, y					
15-19	3830 (0.9)	1136 (1.0)	120 (0.7)	245 (1.0)	2329 (0.9)
20-24	30,661 (7.3)	8794 (7.6)	975 (6.0)	1830 (6.8)	19,062 (7.2)
25-29	108,197 (25.6)	29,236 (25.4)	3817 (23.3)	6750 (25.0)	68,394 (26.0)
30-34	171,784 (40.7)	44,550 (38.7)	6591 (40.3)	10,726 (39.8)	109,917 (41.7)
35-39	91,818 (21.8)	28,086 (22.7)	4056 (24.8)	6155 (22.8)	55,521 (21.1)
40+	14,954 (3.5)	5170 (4.5)	764 (4.7)	1202 (4.5)	7818 (3.0)
Missing	804 (0.2)	212 (0.2)	48 (0.3)	63 (0.2)	481 (0.2)
Birth hospital region					
Eastern Ontario	59,985 (14.2)	12,489 (10.8)	1841 (11.3)	3030 (11.2)	42,625 (16.2)
Central Ontario	159,154 (37.7)	44,199 (38.4)	7629 (46.6)	11,735 (43.5)	95,591 (36.3)
Metro Toronto	118,304 (28.0)	40,324 (35.0)	4348 (26.6)	8360 (31.0)	65,272 (24.8)
Southwestern Ontario	70,247 (16.6)	15,666 (13.6)	2173 (13.3)	3182 (11.8)	49,226 (18.7)
Northern Ontario	14,357 (3.4)	2505 (2.2)	380 (2.3)	684 (2.5)	10,808 (4.1)
Rural residence					
No	373,472 (88.5)	106,035 (92.1)	14,816 (90.5)	24,776 (91.9)	227,845 (86.5)
Yes	44,072 (10.4)	7691 (6.7)	1409 (8.6)	1925 (7.1)	33,047 (12.5)
Missing	4504 (1.1)	1458 (1.3)	146 (0.9)	270 (1.0)	2630 (1.0)

© 2025 Mildon A et al. *JAMA Pediatr*.

eTable 2. Participant characteristics by infant formula supplementation status (continued)

	TOTAL (n=422,048)	Non-medically indicated supplementation (n=115,184)	Medically indicated supplementation (n=16,371)	Missing reason for supplementation (n=26,971)	No supplementation (n=263,522)
Multiparity (≥1 previous birth)					
No	344,545 (81.6)	91,927 (79.8)	14,001 (85.5)	22,959 (85.1)	215,658 (81.8)
Yes	76,305 (18.1)	22,772 (19.8)	2334 (14.3)	3934 (14.6)	47,265 (17.9)
Missing	1198 (0.3)	485 (0.4)	36 (0.2)	78 (0.3)	599 (0.2)
**Maternal Health Characteristics, No. (%)**					
Pre-pregnancy Body Mass Index, kg/m^2					
<18.5	66,089 (15.7)	21,833 (19.0)	2458 (15.0)	5165 (19.2)	36,633 (13.9)
18.5-24.9	195,039 (46.2)	47,128 (40.9)	6694 (40.9)	10,389 (38.5)	130,828 (49.7)
25.0-29.9	93,213 (22.1)	25,656 (22.3)	3843 (23.5)	6081 (22.6)	57,633 (21.9)
30.0-34.9	40,042 (9.5)	11,825 (10.3)	1845 (11.3)	2971 (11.0)	23,401 (8.9)
>35	27,665 (6.6)	8742 (7.6)	1531 (9.4)	2365 (8.8)	15,027 (5.7)
Any pre-existing health condition					
No	334,018 (79.1)	91,235 (79.2)	12, 407 (75.8)	19,960 (74.0)	210,416 (79.9)
Yes	88,030 (20.9)	23,949 (20.8)	3964 (24.2)	7011 (26.0)	53,106 (20.2)
Any mental health concern					
No	347,478 (94.1)	97,893 (85.0)	13,138 (80.3)	21,921 (81.3)	214,526 (81.4)
Yes	69,204 (16.4)	15,854 (13.8)	3046 (18.6)	4569 (16.9)	45,735 (17.4)
Missing	5366 (1.3)	1437 (1.3)	187 (1.1)	481 (1.8)	3261 (1.2)
Smoking at time of birth					
No	397,055 (94.1)	107,718 (93.5)	15,522 (94.8)	25,349 (94.0)	248,466 (94.3)
Yes	16,968 (4.0)	4871 (4.2)	583 (3.6)	1040 (3.9)	10,474 (4.0)
Missing	8025 (1.9)	2595 (2.3)	266 (1.6)	582 (2.2)	4582 (1.7)
Drug use during pregnancy					
No	412,113 (97.6)	112,299 (97.5)	15,982 (97.6)	26,245 (97.3)	257,587 (97.8)
Yes	1518 (0.4)	438 (0.4)	83 (0.5)	171 (0.6)	826 (0.3)
Missing	8417 (2.0)	2447 (2.1)	306 (1.9)	555 (2.1)	5109 (1.9)
Cannabis use during pregnancy					
No	409,135 (96.9)	111,534 (96.8)	15,859 (96.9)	26,016 (96.5)	255,726 (97.0)
Yes	6956 (1.6)	1795 (1.6)	311 (1.9)	513 (1.9)	4337 (1.7)
Missing	5957 (1.4)	1855 (1.6)	201 (1.2)	442 (1.6)	3459 (1.3)

© 2025 Mildon A et al. *JAMA Pediatr.*

eTable 2. Participant characteristics by infant formula supplementation status (continued)

	TOTAL (n=422,048)	Non-medically indicated supplementation (n=115,184)	Medically indicated supplementation (n=16,371)	Missing reason for supplementation (n=26,971)	No supplementation (n=263,522)
**Perinatal Characteristics and Care Provision, No. (%)**					
Antenatal care provider					
Doctor	353,213	105,778 (91.8)	13,960 (85.3)	23,927 (88.7)	209,548 (79.5)
Midwife	45,617	5246 (4.6)	1455 (8.9)	1561 (5.8)	37,355 (14.2)
Other	542	130 (0.1)	16 (0.1)	36 (0.13)	360 (0.1)
Shared care	18,561	2887 (2.5)	854 (5.2)	978 (3.6)	13,842 (5.3)
Missing	4115	1143 (1.0)	86 (0.5)	469 (1.7)	2417 (0.9)
Study year of birth[b]					
Y1	66,304	15,482 (13.4)	2442 (14.9)	4077 (15.1)	44,303 (16.8)
Y2	69,685	17,936 (15.6)	2794 (17.1)	3266 (12.1)	45,689 (17.3)
Y3	69,389	19,987 (16.5)	2651 (16.2)	3208 (11.9)	44,543 (16.9)
Y4	71,432	20,031 (17.4)	2864 (17.5)	3779 (14.0)	44,758 (17.0)
Y5	72,805	19,692 (17.1)	3001 (18.3)	6105 (22.6)	44,007 (16.7)
Y6	72,433	23,056 (20.0)	2619 (16.0)	6536 (24.2)	40,222 (15.3)
Mode of delivery					
Vaginal	302,685	71,384 (62.0)	9454 (57.8)	15,559 (57.7)	206,288 (78.3)
C-section	119,273	43,759 (38.0)	6912 (42.2)	11,403 (42.3)	57,199 (21.7)
Missing	90	41 (0.04)	<6 (S)	9 (0.03)	35 (0.01)
Gestational age at birth, weeks					
37-38	119,457	37,062 (32.2)	6125 (37.4)	9707 (36.0)	66,563 (25.3)
39-40	250,872	66,383 (57.6)	8473 (51.8)	14,459 (53.6)	161,557 (61.3)
41	50,369	11,519 (10.0)	1706 (10.4)	2733 (10.1)	34,411 (13.1)
≥42	1350	220 (0.2)	67 (0.4)	72 (0.3)	991 (0.4)
Infant birth weight, g					
2500-3999	380,071	103,958 (90.3)	13,925 (85.1)	23,428 (86.9)	238,760 (90.6)
4000-4500	36,697	9660 (8.4)	2023 (12.4)	2886 (10.7)	22,128 (8.4)
>4500	5280	1566 (1.4)	423 (2.6)	657 (2.4)	2634 (1.0)
Infant sex					
Female	206,894	56,353 (48.9)	7592 (46.4)	12,360 (45.8)	130,589 (49.6)
Male	215,016	58,802 (51.1)	8773 (53.6)	14,610 (54.2)	132,831 (50.4)
Missing	138	29 (0.03)	6 (0.04)	<6 (S)	102 (0.04)

© 2025 Mildon A et al. *JAMA Pediatr*.

eTable 2. Participant characteristics by infant formula supplementation status (continued)

	TOTAL (n=422,048)	Non-medically indicated supplementation (n=115,184)	Medically indicated supplementation (n=16,371)	Missing reason for supplementation (n=26,971)	No supplementation (n=263,522)
Neonatal Intensive Care Unit admission					
No	399,592	110,130 (95.6)	14,465 (83.4)	17,975 (66.7)	257,022 (97.5)
Yes	22,456	5054 (4.4)	1906 (11.6)	8996 (33.4)	6500 (2.5)
Intention to breastfeed					
No	3237	1805 (1.6)	81 (0.5)	280 (1.0)	1071 (0.4)
Yes	403,495	108,458 (94.2)	15,850 (96.8)	24,200 (89.7)	254,987 (96.8)
Unknown/unsure	1936	903 (0.8)	62 (0.4)	138 (0.5)	833 (0.3)
Missing	13,380	4018 (3.5)	378 (2.3)	2353 (8.7)	6631 (2.5)
Skin-to-skin contact $\geq$ 1 hour in first 2 hours after birth					
No	15,563	4766 (4.1)	921 (5.6)	4863 (18.0)	5113 (1.9)
Yes	343,444	92,286 (80.1)	11,762 (71.9)	17,941 (66.5)	221,455 (84.0)
Missing	62,941	18,132 (15.7)	3688 (22.5)	4167 (15.5)	36,954 (14.0)
Breastfeeding initiation within 2 hours of birth					
No	9827	3283 (2.9)	544 (3.3)	2347 (8.7)	3653 (1.4)
Yes	300,981	78,019 (67.7)	11,720 (71.6)	14,250 (52.8)	196,992 (75.5)
Missing	111,240	33,882 (29.4)	4107 (25.1)	10,374 (38.5)	62,877 (23.9)
Postpartum breastfeeding support provided					
No	4779	1466 (1.3)	90 (0.6)	905 (3.4)	2318 (0.9)
Yes	395,284	107,358 (93.2)	15,516 (94.8)	23,566 (87.4)	248,844 (94.4)
Missing	21,985	6360 (5.5)	765 (4.7)	2500 (9.3)	12,360 (4.7)

S=data suppressed due to low cell count. [a]Other racial group includes individuals of Indigenous, mixed and unknown race. [b]Y1: April 1, 2015-March 31, 2016; Y2: April 1, 2016-March 31, 2017; Y3: April 1, 2017-March 31, 2018; Y4: April 1, 2018-March 31, 2019; Y5: April 1, 2019-March 31, 2020; Y6: April 1, 2020-March 31, 2021.

© 2025 Mildon A et al. *JAMA Pediatr*.

eTable 3. Sub-group analysis of associations between maternal socio-economic marginalization quintiles and non-medically indicated formula supplementation, comparing births in study years prior to the COVID-19 pandemic (Y1-5) and in the first year of the pandemic (Y6)

	**Frequency,** n (%)		**Unadjusted RR** (95% CI)		**Model 1 Adjusted RR** (95% CI)		**Model 2 Adjusted RR** (95% CI)	
**Quintile**	**Y1-5 (n=320,172)**	**Y6 (n=64,484)**	**Y1-5**	**Y6**	**Y1-5**	**Y6**	**Y1-5**	**Y6**
Quintile 1	11,889 (3.7)	3094 (4.8)	1 (ref)	1 (ref)	1 (ref)	1 (ref)	1 (ref)	1 (ref)
Quintile 2	17,500 (5.5)	4709 (7.3)	1.29 (1.25-1.32)	1.32 (1.25-1.39)	1.17 (1.14-1.20)	1.21 (1.14-1.28)	1.16 (1.13-1.19)	1.22 (1.15-1.29)
Quintile 3	18,959 (5.9)	4852 (7.5)	1.55 (1.51-1.59)	1.57 (1.49-1.66)	1.34 (1.30-1.38)	1.33 (1.26-1.41)	1.31 (1.27-1.35)	1.34 (1.26-1.42)
Quintile 4	18,052 (5.6)	4622 (7.2)	1.78 (1.74-1.83)	1.77 (1.68-1.88)	1.52 (1.48-1.57)	1.49 (1.41-1.58)	1.46 (1.42-1.51)	1.47 (1.38-1.56)
Quintile 5	22,978 (7.2)	5340 (8.3)	2.24 (2.18-2.30)	2.18 (2.07-2.30)	1.81 (1.76-1.86)	1.75 (1.65- 1.85)	1.69 (1.66-1.74)	1.68 (1.58-1.78)

R=Relative Risk. CI=Confidence Interval. Y1-5: April 1, 2015-March 31, 2020; Y6: April 1, 2020-March 31, 2021. Quintile 1=least marginalized. Model 1 adjusted for maternal socio-demographics (age, race, rural residence, birth hospital region, parity). Model 2 adjusted for Model 1 covariates and maternal health characteristics (pre-pregnancy BMI, pre-existing health conditions, mental health concerns, prenatal smoking, prenatal substance use, prenatal cannabis use).

© 2025 Mildon A et al. *JAMA Pediatr.*

eTable 4. Sub-group analysis of associations between maternal race and non-medically indicated formula supplementation, comparing births in study years prior to the COVID-19 pandemic (Y1-Y5) and in the first year of the pandemic (Y6)

	**Frequency,** n (%)		**Unadjusted RR** (95% CI)		**Model 1 Adjusted RR** (95% CI)		**Model 2 Adjusted RR** (95% CI)	
**Race**	**Y1-5 n=326,962**	**Y6 n=65,762**	**Y1-5**	**Y6**	**Y1-5**	**Y6**	**Y1-5**	**Y6**
Asian	36,882 (11.3)	10,174 (15.5)	2.79 (2.74-2.83)	3.39 (3.27-3.51)	2.47 (2.43-2.52)	2.82 (2.71-2.94)	2.63 (2.57-2.68)	2.91 (2.78-3.03)
Black	8937 (2.7)	2209 (3.4)	2.60 (2.60- 2.76)	2.64 (2.48-2.81)	2.09 (2.03-2.16)	1.97 (1.84-2.10)	2.09 (2.02-2.15)	1.93 (1.80-2.06)
Other	4988 (1.5)	1113 (1.7)	1.60 (1.55-1.66)	1.47 (1.36-1.58)	1.44 (1.39-1.49)	1.33 (1.23-1.44)	1.46 (1.40-1.51)	1.33 (1.23-1.44)
White	40,592 (12.4)	9504 (14.5)	1 (ref)	1 (ref)	1 (ref)	1 (ref)	1 (ref)	1 (ref)

RR=Relative Risk. CI=Confidence Interval. Y1-5: April 1, 2015-March 31, 2020; Y6: April 1, 2020-March 31, 2021. Model 1 adjusted for maternal socio-demographics (age, ON-Marg quintile, rural residence, birth hospital region, parity). Model 2 adjusted for Model 1 covariates and maternal health characteristics (pre-pregnancy BMI, pre-existing health conditions, mental health concerns, prenatal smoking, prenatal substance use, prenatal cannabis use).

© 2025 Mildon A et al. *JAMA Pediatr*.

eTable 5. Associations between maternal socio-economic marginalization quintiles and non-medically indicated formula supplementation, adjusted for maternal socio-demographics, maternal health characteristics and perinatal characteristics (n=384,656)

Quintile	Frequency, n (%)	Unadjusted RR (95% CI)	Adjusted RR (95% CI)
Quintile 1	14,983 (3.9)	1 (ref)	1 (ref)
Quintile 2	22,209 (5.8)	1.29 (1.26-1.32)	1.18 (1.15-1.23)
Quintile 3	23,811 (6.2)	1.55 (1.52-1.59)	1.31 (1.26-1.35)
Quintile 4	22,674 (5.9)	1.78 (1.74-1.83)	1.42 (1.37-1.47)
Quintile 5	28,318 (7.4)	2.22 (2.17-2.27)	1.66 (1.60-1.72)

RR=relative risk. CI=Confidence Interval. Covariates: maternal socio-demographics (age, race, rural residence, birth hospital region, parity), maternal health characteristics (pre-pregnancy BMI, pre-existing health conditions, mental health concerns, prenatal smoking, prenatal substance use, prenatal cannabis use) and perinatal characteristics (antenatal care provider, study year of birth, mode of delivery, gestational age at birth, infant birth weight, infant sex, NICU admission, intention to breastfeed, skin-to-skin contact, timely breastfeeding initiation and provision of postpartum breastfeeding support).

© 2025 Mildon A et al. *JAMA Pediatr*.

eTable 6. Associations between maternal race and non-medically indicated formula supplementation, adjusted for maternal socio-demographics, maternal health characteristics and perinatal characteristics (n=316,214)

Race	Frequency, n (%)	Unadjusted RR (95% CI)	Adjusted RR (95% CI)
Asian	47,056 (14.9)	2.89 (2.85-2.94)	2.80 (2.73-2.87)
Black	11,146 (35.2)	2.68 (2.61-2.75)	2.08 (2.00-2.16)
White	50,096 (15.8)	1 (ref)	1 (ref)
Other	6101 (1.9)	1.58 (1.53-1.63)	1.49 (1.42-1.55)

RR=relative risk. CI=Confidence Interval. Covariates: maternal socio-demographics (age, race, rural residence, birth hospital region, parity), maternal health characteristics (pre-pregnancy BMI, pre-existing health conditions, mental health concerns, prenatal smoking, prenatal substance use, prenatal cannabis use) and perinatal characteristics (antenatal care provider, study year of birth, mode of delivery, gestational age at birth, infant birth weight, infant sex, NICU admission, intention to breastfeed, skin-to-skin contact, timely breastfeeding initiation and provision of postpartum breastfeeding support).

© 2025 Mildon A et al. *JAMA Pediatr*.
