# Supplementary material for: Hospital Formula Supplementation Postbreastfeeding Initiation, Neighborhood Economy, and Race
Source: JAMA Pediatr. 2025 Dec 29;180(3):263–74. doi: 10.1001/jamapediatrics.2025.5379 (PMC12750333; doi:10.1001/jamapediatrics.2025.5379)
Supplement: Supplement 2. — Data sharing statement [file jamapediatr-e255379-s002.pdf]

Data Sharing Statement

Mildon. Hospital Formula Supplementation Postbreastfeeding Initiation, Neighborhood Economy, and Race. *JAMA Pediatr*. Published December 29, 2025. doi:10.1001/jamapediatrics.2025.5379

Data
Data available: No

Additional Information
Explanation for why data not available: The dataset from this study is held securely by BORN Ontario and cannot be made publicly available due to privacy laws and the Personal Health Information Protection Act in Ontario, Canada.
